# TIGAR deficiency induces caspase-1-dependent trophoblasts pyroptosis through NLRP3-ASC inflammasome

**DOI:** 10.3389/fimmu.2023.1114620

**Published:** 2023-04-14

**Authors:** Junjun Guo, Meijuan Zhou, Man Zhao, Shuxian Li, Zhenya Fang, Anna Li, Meihua Zhang

**Affiliations:** Key Laboratory of Birth Regulation and Control Technology of National Health Commission of China, Maternal and Child Health Care Hospital of Shandong Province Affiliated to Qingdao University, Jinan, Shandong, China

**Keywords:** TIGAR, High glucose, trophoblast, pyroptosis, ROS, NLRP3 inflammasome

## Abstract

**Introduction:**

Gestational diabetes mellitus (GDM), a common complication of pregnancy, is risky for both mother and fetus. Previous studies about TP53-induced glycolysis and apoptosis regulator (TIGAR) focused on the occurrence and development of cancer, cardiovascular disease, and neurological disease, however, it is still unclear whether TIGAR plays a regulatory role in gestational diabetes mellitus (GDM).

**Methods:**

Utilizing HG exposure, we explored the role of TIGAR in oxidative stress limitation, excessive inflammatory toxicity defense, and pyroptosis prevention.

**Results:**

TIGAR was up-regulated *in vivo* and *in vitro* under HG condition, and loss of TIGAR increased ROS in trophoblast cells which drove a phenotypic switch and hindered the capacity of migration, invasion, and tube formation. This switch depended on the increased activation of NLRP3-ASC-caspase-1 signaling, which caused a distinctive characteristic of pyroptosis, and these findings could finally be reverted by antioxidant treatment (NAC) and receptor block (MCC950). Collectively, trophoblast pyroptosis is an upstream event of TIGAR deficiency-induced inflammation, which is promoted by ROS accumulation through NLRP3-ASC inflammasome.

**Conclusion:**

Taken together, our results uncovered that, as the upstream event of TIGAR deficiency-induced inflammation, pyroptosis is stimulated by ROS accumulation through NLRP3-ASC inflammasome.

## Introduction

Gestational diabetes mellitus (GDM) is the most common complication of pregnancy, which is defined as “the type of glucose intolerance with onset or first recognition during pregnancy” (1–3). GDM is caused by insulin resistance and dysfunction of pancreatic β-cell during pregnancy, resulting in hyperglycemia of variable severity, including cesarean delivery, shoulder dystocia, macrosomia, neonatal hypoglycemia, cardiovascular diseases and type 2 diabetes mellitus (T2DM) both in the mother and her offspring (4, 5). The etiology of GDM is closely related to obesity, environment, and genetic factors (6). Despite considerable progress has been made in the early detection and treatment of diabetes in the past decades, GDM can still led to fetal growth disorder even when an optimized and intensive metabolic control has been achieved (7, 8). Therefore, to explore effective approaches for prevention, early screening, diagnosis and manage of GDM is of vital essence.

There is a direct correlation between GDM and dysfunction of glucose and lipid metabolism involving multiple genes. TP53-induced glycolysis and apoptosis regulator (TIGAR), a novel p53-inducible protein, was first discovered in 2006 (9, 10). TIGAR is characterized as a fructose-2, 6-bisphosphatase that can support the activation of the oxidative pentose phosphate pathway (PPP) to reduce glycolysis and protect against oxidative stress by augmenting of NADPH (11, 12). The functions of TIGAR to limit ROS (Reactive oxygen species) has been shown to be helpful to the solution of ischemia/reperfusion and protect against pathologies such as ischemia disease (13). However, little is known about the role of TIGAR in GDM. In this study, we aimed to investigate the effect of TIGAR on GDM placenta injury and explore the underlying molecular mechanism.

ROS contributes a dual role to the development of disease that function in cells as signaling molecules and unavoidable toxic byproducts of aerobic metabolism (14, 15). Numerous studies support the concept that ROS is involved in the initiation and development of diseases. Mechanically, ROS play important and diverse roles in regulating various cellular behavior, such as proliferation, invasion, migration and viability (16, 17). ROS can induce both genotoxic damage and chronic inflammation, while membrane-associated ROS generated through NADPH oxidases (such as NOX4) is an important contributor to the activation of signaling pathways that drive proliferation and metastasis (18, 19). Mitochondria is the main source of ROS generation as well as the major target organelle of free radical attack. ROS generated by NADPH oxidase was shown to stimulate mitochondrial ROS production and induce mitochondrial dysfunction (20, 21). Furthermore, mitochondrial ROS was also shown to be necessary for cancer development and many studies previously demonstrated that mitochondrial ROS plays a key role in aging and lifespan regulation (22, 23). Therefore, out of control of ROS production and failure of antioxidant defense have been associated with many aspects of human health and disease. Many mechanisms that regulate ROS exposure have been studied. As a transcription factor regulating antioxidant defense, the activation of NRF2 is the most ubiquitous (24, 25). Another protein that contributes to ROS limitation is TIGAR (26), nevertheless, the mechanism in early and later stages of GDM development still remains a terra incognita.

Disturbed immune homeostasis and excessive oxidative stress cause abnormal trophoblast differentiation and invasion which underpin placental-based pregnancy complications. As a new-found programmed cell death involving proinflammatory and autolytic, pyroptosis has been proved to play an crucial role in the pathogenesis of various pregnancy complications (27, 28). Excessive activation of NLRP3 inflammasome, which consist of NOD-like receptor pyrin domain-containing protein 3 (NLRP3), apoptosis associated speck-like protein containing CARD (ASC), and Pro-cysteinyl aspartate specific proteinase-1 (Pro-caspase-1), is involved in the pathogenesis of maternal-fetal interface disorders (29, 30). Within the cell, execrable and constant stimulus such as excessive synthesis of ROS activate pyroptosis priming, with the activation of NLRP3 inflammasome. Such as a proteolytic enzyme, NLRP3 inflammasome hydrolyzes Pro-caspase-1 to form cleaved caspase-1 and slices Gasdermin D (GSDMD). Several P30 subunits generated from the N-terminal of GSDMD polymerize on the plasma membrane and generate a non-selective ion channel, which confers the maturation and release of proinflammatory cytokines [such as Pro-interleukin-1β (Pro-IL-1β), interleukin-6 (IL-6), and interleukin-18 (IL-18)], ultimately pyroptosis is induced (31, 32). Cleaved caspase-1 converts the cytokine precursors pro-IL-1β and pro-IL-18 into mature and biologically active IL-1β and IL-18, respectively (33, 34). IL-1β activation plays a central role in mediating the proinflammatory response, which results in the initiation of secondary inflammatory mediators, including IL-6, TNF-α, IFNγ, et al (35, 36). In turn, IL-6 acts detrimentally in the elicitation of acute phase response with hepatic production of acute phase proteins, such as fibrinogen, C-reactive protein (CRP), and plasminogen activator inhibitor (37). IL-18 is biologically and structurally related to IL-1β, which is important for the production of interferon-γ and potentiation of cytolytic activity of natural killer cells and T cells (38). Therefore, IL-1 cytokines and particularly IL-1β attract most attention which display as valuable targets in the treatment of inflammatory diseases.

In this study, we investigated the role of TIGAR in the initiation and development of HG-induced pyroptosis. At the beginning, TIGAR was enhanced in GDM placenta. Using established HG model in mice and trophoblast, we observed elevated expression of TIGAR. Subsequently, excess generation and release of ROS were discovered upon loss of TIGAR, accompanied by decreased capacity of migration and invasion. Consistent with the above effects, a distinctive characteristic of pyroptosis and NLRP3-ASC inflammasome were described. The responses to TIGAR deletion were plastic and reverted by treatment of cells with antioxidants NAC. Consistently, the pattern of TIGAR expression in both placenta and trophoblasts suggested the role in ROS limitation.

## Methods

### Cell culture and treatment

Immortalized human chorionic trophoblast line HTR-8/Svneo was purchased from ATCC, and cultured in RPMI-1640 medium supplemented with 10% fetal bovine serum (FBS; Gibco, New Zealand) in a humidified incubator with 5% CO_2_ in air atmosphere at 37°C. The cells were passaged every 2~3 days and were harvested at the logarithmic phase. Primary extravillous trophoblasts (EVTs) were isolated as previously described (39).

D-glucose was obtained from Sigma-Aldrich. The cells were cultured in the respective medium supplemented with high glucose (HG) (30 mM D-glucose) and the control (5.5 mM D-glucose, as physiological concentration) throughout the whole culture process. Subsequently, cells were transfected with siRNA to knockdown TIGAR efficiently. After successful gene-editing, cells were co-treated with inhibitors or other stimulating agents for different intervention in the preceding condition(5.5mM or 30mM), and then were harvested at 48h for various detection.

### Human subjects

This study was approved by the ethics committee of Maternal and Child Health Care Hospital of Shandong Province Affiliated to Qingdao University, and informed consent was obtained from all subjects. We included 6 women with GDM and 6 age-matched control subjects at the time of cesarean section. The placental tissue was used to detect the expression of TIGAR. The diagnostic criteria of GDM were as follows: fasting blood glucose >5.1 mmol/L, and/or 1-h glucose >10.0 mmol/L, and/or 2-h glucose>8.5 mmol/L. The inclusion criteria of participants were as follows: age≥20 years, firstborn single pregnancy, no previous diagnosis of diabetes, no history of medications affecting glucose metabolism, no infectious diseases, and no clinically significant neurological, endocrinological, or other systemic disease. All participants were recruited from Maternal and Child Health Care Hospital of Shandong Province Affiliated to Qingdao University. The clinical information was shown in **Table 1**.

**Table 1:** Clinical information of participator with or without GDM.

Characteristics	Control(n=6)	GDM(n=6)	P-value
Age, years	30.33±4.33	31.83±4.1	<0.0001
BMI (Pre-pregnancy BMI, kg/m^2^)	24.86±3.05	28.01±3.66	<0.001
Family history of diabetes (%)	38.6%	45.1%	0.018
History of arterial hypertension (%)	7.7%	8.5%	0.521
History of GDM (%)	0.8%	12.2%	<0.001
History of IGT (%)	2.1%	4.4%	0.031
PCOS (%)	5.8%	7.8%	0.265
Parity:			
Nulliparae (%)	41.8%	33.5%	
Multiparae (%)	58.6%	65.6%	
Number of pregnancies*	1.6±1.4	2.2±1.8	<0.001
Systolic BP (mm Hg)	110.8±151	11.6.8±13.6	<0.001
Diastolic BP (mm Hg)	70.5±8.8	75.1±8.1	<0.001
Fasting plasma glucose (mmol/L)	4.51±0.7	5.2±0.5	<0.001
1-h postload glucose (mmol/L)	7.1±1.3	9.8±1.6	<0.001
2-h postload glucose (mmol/L)	5.8±1.4	7.8±1.5	<0.001
Insulin,mIU/L**	10.4±5.8	14.6±6.2	<0.001
HOMA**	2.1±1.2	3.4±2.6	<0.001

BMI, body mass index; GDM, gestational diabetes mellitus; IGT, impaired glucose tolerance; PCOS, polycystic ovary syndrome; BP, blood pressure. *including the index pregnancay.

**analysis performed in 10 patients with GDM and 10 women from control group.

### Animals

Healthy C57BL/6J male and female mice were purchased from Huachuang Sino (Jiangsu, China) and kept in controlled environment (12h/12h light/dark cycles, 22 ± 1°C) with free access to food and water. After 6 weeks, breeding was conducted overnight in a 1:2 ratio; mating was confirmed by presence of a vagina mucous plug in the following morning, which represented gestation day GD0.5, then the female mice were randomly divided into two groups: GDM and Negative Control (NC). According to many references, we successfully established the GDM model (40–42). Detailly, streptozotocin (110 mg/kg i.p.; Sigma-Aldrich) or equivalent saline was injected on the following 3 days. The blood glucose rose to 20 mM, which marked successful establishment of GDM mice model. The female mice were sacrificed at GD16.5, blood and placenta tissues were collected for subsequent detections. All experimental procedure was approved by Ethical Committee of Maternal and Child Health Care Hospital of Shandong Province Affiliated to Qingdao University. The detailed information that could successful support the GDM model construction was shown in **Supplementary Figure 1**.

### Reagents

Beta Actin (β-Actin) (6600901, proteintech, Wuhan, China) was used as a loading control, Horseradish peroxidase-labeled goat-anti-rabbit immunoglobulin G (GB23303) and Horseradish peroxidase-labeled goat-anti-rabbit immunoglobulin G (GB23301) was the secondary antibody for western blot, both of them were purchased from Servicebio (Wuhan, China). RPMI 1640 medium (L210KJ) and RPMI 1640 medium (-D-glucose) (L270KJ) were purchased from BasalMedia (Shanghai, China). Fetal bovine serum (FBS) (10091148) was obtained from Gibco (USA). Adenosine triphosphate (ATP) (C0550), and mitochondrial membrane potential assay kit (C2006) were purchased from Solarbio (Beijing, China). MCC950 (256373-96-3) and D-(+)-glucose (G6152) were provided by Sigma-Aldrich (MO, USA). Primary antibodies, including TIGAR (ab37910) was purchased from Abcam (UK), Caspase-1 (A0964), NLRP3 (A5652), ASC (A16672), and IL-1β(A19635), were supplied by Abclonal (Wuhan, China), IL-6 (21865-1-AP) and TNF-α (17590-1-AP) were provided by proteintech (Wuhan, China). Cell counting kit 8 (CCK-8) (BA00208) was supplied by Bioss (Beijing, China), lactate dehydrogenase (LDH) assay kit (C0017), JC-1 (C2005) and N-acetyl-L-cysteine (NAC) (ST1546-10g) were purchased from Beyotime (Beijing, China), 6-carboxy-2’,7’-dichlorodihydrofluorescein diacetate di (acetoxymethyl ester) (DCFH-DA) (C2938), Hoechst 33342 (H1399) and MitoSOX™ Red mitochondrial superoxide indicator (M36008) were purchased from Invitrogen (Carlsbad, CA).

### CCK-8 cell viability assay

CCK-8 Cell Viability Assay was used to assess the effects of TIGAR-knockdown on the viability of HTR-8/Svneo cells under high glucose condition. According to the different group processing requirements, 7000 cells/well were seeded onto 96-well plate and divided into seven groups:

### Oxidative stress assessment

After indicated treatment, the cells and culture medium supernatants were collected. Levels of total glutathione (GSH), glutathione disulfide (GSSG) and superoxide dismutase (SOD) activity were detected by kit (Beyotime, Beijing, China).) according to the manufacturer’s instructions. The relative levels were analyzed on the microplate reader (SynergyH1, BioTek, USA).

### Quantitative real-time PCR

Isolation of the total RNA in trophoblast cells was conducted by means of utilizing TRIzol reagent (Invitrogen, USA). 1 μg of total RNA was reverse-transcribed to generate cDNA with M-MLV reverse transcription (TOYOBO CO., LTD. Osaka, Japan), which is then applied to perform qRT-PCR equipped with SYBR green fluorescent dyes (TOYOBO CO., LTD. Osaka, Japan). The primers were obtained from BioSune (Shanghai, China) and the primer sequences were as follows: TIGAR, forward, 5’-ATGGCTCGCTTCGCTCTG-3’, reverse, 5’-CTTCCCTGGCTGCTTTGG-3’; GAPDH, forward, 5’-GGAGCGAGATCCCTCCAAAAT-’3, reverse, 5’-GGCTGTTGTCATACTTCTCATGG-3’. The mRNA level was calculated using 2-^ΔΔCt^ method. GAPDH was used as internal reference.

### LDH release assay

The cytotoxicity was evaluated by detecting the LDH leakage into the culture medium according to the manufacturer’s instructions of Cytoscan-LDH cytotoxicity assay kit (Beyotime, Beijing, China). Briefly, the cells were transferred onto a 96-well plate (7×10^3^ cells/well) which cultured in normal or HG conditions, and transfected with siRNA, then treated with inhibitors or ATP for different periods. After indicated treatment, the culture supernatant was collected and centrifuged at 400 g for 5 min, and the cell debris was discarded. 120 μl supernatant was transferred to a clean 96-well plate, and 60 μl reaction mixture was added to each well for another incubation for 30 min in the dark at room temperature. The absorption at 490 nm was detected using a modular multimode microplate reader (SynergyH1, BioTek, USA).

### Invasion assays

Invasion assays was performed according to the manufacturer’s instructions (Corning, Bio-Coat™ Matrigel Invasion chamber). Briefly, cells were performed with indicated treatments before seeding onto the upper chamber. Media with 5% serum were used in the lower chamber. After 16 hours, cells that remained on the top membrane were removed by a cotton-tipped applicator. Cells that invaded into the bottom of the insert were then fixed in 4% PFA and stained with 0.5% Crystal Violet. Invaded cells were photographed under an inverted microscope, quantified using Image J.

### Wound scratch assay

Confluent monolayer of cells was scratched using a 200μl pipette tip to create a scratch. Debris were removed by washing the cells gently with PBS. Images were taken at the start of the assay and after 48 hours under a phase contrast microscope. The width of the gap was measured by ImageJ and the reduction of the width is represented as percentage (%) wound closure.

### ROS measurement

10μM 2’,7’-dichlorofluorescin diacetate (DCFH-DA, Beyotime, China), a ROS-specific fluorescent probe which is oxidized into fluorescent dichlorofluorescein (DCF), was used to measure total intracellular ROS levels. And mitochondrial ROS was evaluated by the uptake of MitoSox-Red (Molecular Probes, Life Technologies). HTR-8 cells (7×10^3^ cells) were seeded in a 96-well plate, after successful TIGAR-siRNA transfection and compression treatment, cells were detached by trypsinization and then incubated with 100μl 10μM DCFH-DA or 5μM MitoSox-Red dissolved in DMEM without FBS for 30 min at 37°C under dark condition. After washing with serum-free medium three times, the cells were analyzed by flow cytometry (FACS Caliber; Becton Dickinson, Heidelberg, Germany) to detect the mean fluorescence intensity (MFI) with an excitation wavelength of 488 nm and an emission wavelength of 525 nm. Accordingly, the ROS levels were quantified and compared. The fluorescence intensity of specific probe was observed and quantified by the ImageXpress^®^ micro confocal system.

### Mitochondrial transmembrane potential measurement

Mitochondrial membrane potential was estimated by monitoring fluorescence aggregates of JC-1 (Molecular Probes, Invitrogen, UK, T3168). After treated with the designate conditions, HTR-8 was incubated with 2.5 μg/ml JC-1and incubated at 37°C in dark for 20 min. Then removed supernatant and washed twice with pre-cooled JC-1 buffer. Finally, 0.2 mL medium was added and observed with fluorescence microscope.

### Transmission electron microscopy

The ultrastructural features of HTR-8 cells with indicated treatment were examined using TEM electron microscopy (TEM). Briefly, after specific intervention, the cells were fixed with 2.5% glutaraldehyde and collected softly with centrifugation, then washed twice in PBS and fixed in preservation and transportation. Then cells were postfixed for 2 h with 1% OsO4, followed by dehydration with a series of concentrations of ethanol (30%, 50%, 70%, 80%, 85%, 90%, 95%, 100%), and then infiltration and embedding in epoxy resin, which were cut into slices of 60-80nm subsequently. Finally, ultrathin sections were stained with uranyl acetate and lead citrate and subjected to transmission electron microscope (TEM) for images taken.

### Western blot analysis

A total of 3×10^5^ HTR-8 cells/well were plated onto a 6-wellplate and treated with specified interventions. Protein lysates were extracted in RIPA-buffer (Solarbio, Beijing, China) containing proteinase inhibitor cocktail (Beyotime, Beijing, China) and quantified using BCA kit (Solarbio, Beijing, China). Equal protein were resolved in SDS-PAGE and electrophoretically transferred onto polyvinylidene fluoride (PVDF) membranes by wet method. After blocking in 5% non-fat milk for 1 h at room temperature, the membranes were incubated with diluted primary antibodies overnight at 4°C. On the following day, the membranes were rinsed for 3 times with TBST, and then hybridized with horseradish peroxidase-labeled secondary antibody for1 h at room temperature. After treating with an enhanced chemiluminescence detection kit (Amersham LifeScience, Buckinghamshire, UK), the bands were detected by Gel Imager System. The expression of target proteins was quantified by normalizing to β-actin. Independent experiments were performed at least three times, and the representative pictures were presented.

### Measurement of IL-1β and IL-6

Pro-inflammatory cytokine concentration of IL-1β in the cell culture supernatant was determined by using the Human IL-1β enzyme-linked immunosorbent assay (ELISA) Kit (EK0392, BOSTER, Wuhan, China) following the supplier’s protocol.

IL-6 ELISA Kit (EK0411, BOSTER, Wuhan, China).

### Transfection of siRNA

Knockdown of TIGAR was achieved by transfecting TIGAR-siRNA fragment (Gene Pharma, shanghai, China) with the corresponding scrambled siRNA as negative control. Transfection was performed using Lipofectamine RNAiMAX for siRNA (Invitrogen) according to the manufacturer’s instructions.

The sequences of small interfering RNAs (siRNAs)

TIGAR Sence:5’-GCUUUGUAUCCAAGCUGCCUGUGAA-3’

TIGAR Antisense: 5’- UUCACAGGCAGCUUGGAUACAAAGC-3’

### Immunofluorescence staining

Cells were fixed in 4% paraformaldehyde, followed by permeabilization with 0.1% Triton X-100 in 1×PBS, then blocking with 10% normal goat serum for 1h in the room temperature. We performed dewaxing, hydration, and antigen repair on paraffin sections of placentas, then blocked as same as the cells preparation. Primary antibody was prepared in 1×PBS with 0.1% Triton X-100 and 10% normal goat serum. Antibody used were TIGAR (at 1µg/ml, Abcam, ab37910) and CK-7(1:200, Abcam, ab9021). Blocked cells were incubated overnight at 4°C with the primary antibody, washed 3× in 1×PBS, followed by incubation of secondary antibody for 1 hour at room temperature (1:100 in 1×PBS with 0.1% Triton X-100 and 5% normal donkey serum, Alexa Fluor 488 goat anti-rabbit and Tritc goat anti-mouse, Servicebio). Antibody used in negative control were anti-human IgG (ab109489) and anti-mouse IgG (ab190475).

### Statistical analysis

Data are presented as the mean ± SEM. For experiments comparing two groups of data, unpaired Student’s t-test was performed. For data involving multiple groups (≥3), one‐way analysis of variance (ANOVA) was performed followed by an SNK *post-hoc* test. The quantified results were visualized using bar charts, p values<0.05 were considered significant.

## Results

### TIGAR is upregulated under HG condition

**Figure 1:**
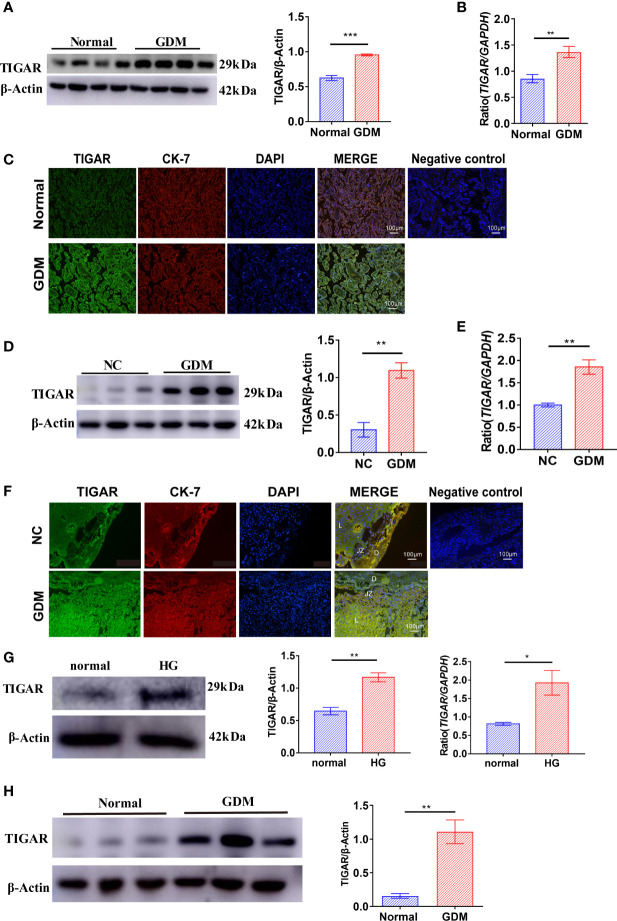
TIGAR levels were upregulated in the placenta of patients and mice with GDM and HTR-8 cells cultured with high glucose. **(A)** Immunoblotting for TIGAR in placental lysates from patients with GDM*(n=4)* or Normal patients*(n=4).* **(B)** q-PCR analysis of TIGAR in placental tissues from patients with GDM*(n=6)* or Normal patients*(n=6).* **(C)** Immunostaining for TIGAR on the free villous (FV) of placental sections from patients with GDM*(n=4)* or Normal patients*(n=4).* Negative control for the Immunofluorescent staining of TIGAR expression was on the right. Scale bar=100μm. **(D)** Immunoblotting for TIGAR in placental lysates from GDM mice*(n=3)* or NC*(n=3).* **(E)** q-PCR analysis of TIGAR in placental tissues of GDM mice*(n=6)* or NC*(n=6).* **(F)** Immunostaining for TIGAR on the labyrinth zone (Lz) of placental sections from GDM mice *(n=4)* or NC*(n=4)* and we illustrated the structure of placenta, as Decideua (De), labyrinth zone (Lz), junctional zone (Jz) in the images, negative control for the Immunofluorescent staining of TIGAR expression was on the right. Scale bar=100μm. Immunoblotting **(G)** and q-PCR analysis **(H)** for TIGAR in HTR-8 which cultured in 5.5mM normal glucose and 30mM high glucose (HG) (n=3). *p < 0.05, **p < 0.01, ***p < 0.001 versus the indicated group. TIGAR, TP53 induced glycolysis regulatory phosphatase.

Firstly, we evaluated TIGAR expression in human placenta of GDM patients (n=4) or Normal patients (n=4). Compared with controls, the expression of TIGAR in placenta of GDM patients was significantly upregulated (**Figures 1A, B** **)**, as detected by western blotting and q-PCR. Immunofluorescence results showed that expression of TIGAR increases on the free villous (FV) of GDM patients (**Figure 1C**). As shown in **Figures 1D, E**, the protein and mRNA expression of TGAR in mice placenta with GDM increased prominently. Notably, TIGAR level was specifically elevated in the labyrinth (L) zone of GDM mice (**Figure 1F**). To further verify the above results, we cultured HTR-8 cells under high glucose(30mM) and control (5.5mM) conditions. Undoubtedly, HG exposure elevated TIGAR expression both on protein and RNA levels (**Figure 1G**). And in primary extravillous trophoblasts (EVTs) isolated from placentas of Normal or GDM patients, TIGAR was also upregulated (**Figure 1H**). Collectively, these data indicated that TIGAR conserved a vital effect in the defense of HG-induced impairment.

### TIGAR deficient HTR-8/Svneo cells are highly sensitive to high glucose impairment

**Figure 2:**
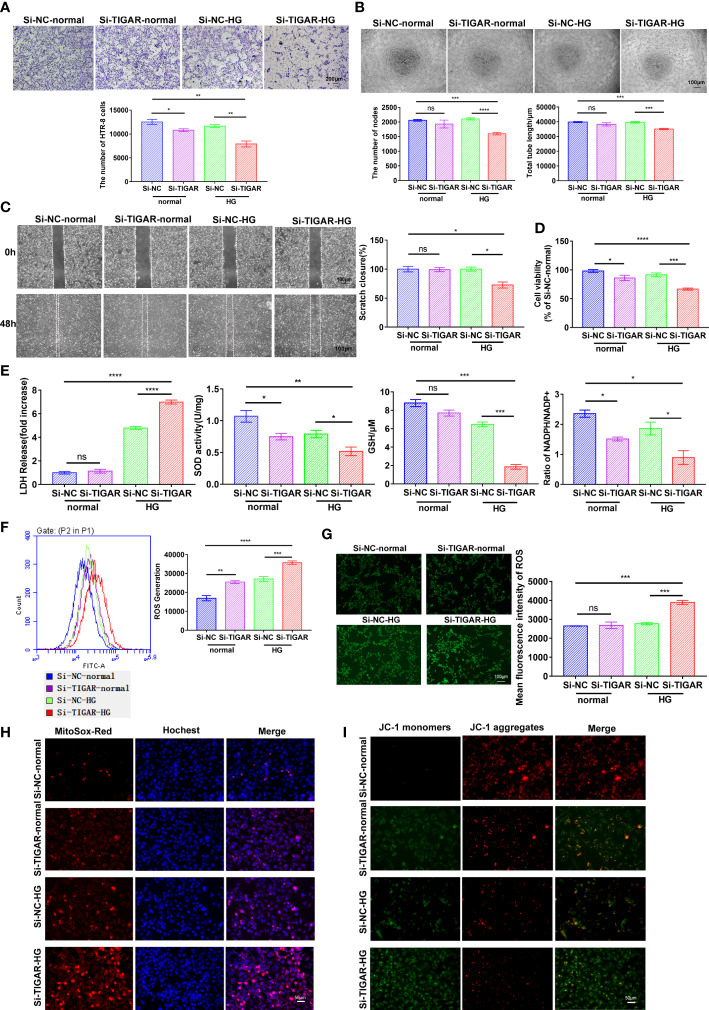
The effect of TIGAR loss on the viability and biological function of HTR-8/HTR-8/Svneo. **(A)** Representative images for invasion test and quantification of the number of cells per field (x100). Scale bar: 200μm. **(B)** Representative images for tube formation test and quantification of the number of nodes and tube length. Scale bar: 100μm. **(C)** Representative images for scratch test and quantification of wound healing area. Scale bar: 100μm. **(D)** Cell viability of HTR-8 treated with normal or HG, which was transfected with TIGAR‐siRNA (Si-TIGAR) or Negative control‐siRNA (Si-NC). **(E)** LDH release, SOD activity, GSH level and ratio of NADPH/NADP^+^ were measured at 48h after specific stimulus. All experiments were from at least three independent experiments. Data were expressed as mean ± SEM. Intracellular ROS levels in Si-TIGAR and Si-NC HTR-8 when treated under normal or HG condition were determined using DCFH-DA, as detected by flow cytometry **(F)** and photograph **(G)** The histogram shows the mean ROS production. Scale bar: 100μm. Mitochondrial superoxide was evaluated by JC-1 staining **(H)** and MitoSOX Red staining **(I)** of HTR-8 cells. Scale bar: 50μm. Data of the triple experiments are presented as the means ± SEM. *p < 0.05, **p < 0.01, ***p < 0.001, ****p < 0.0001 versus the indicated group, ns, no significance. GSH, glutathione; GSSG, oxidized glutathione; NADP+, nicotinamide adenine dinucleotide phosphate; NADPH, reduced NADP+; ROS, reactive oxygen species.

Herein, to investigate the effects of TIGAR on GDM, we successfully interfered the expression of TIGAR with siRNA. As illustrated in **Figure 2**, TIGAR deficiency significantly inhibited the abilities of cell invasion **(** **Figure 2A** **)**, tube formation **(** **Figure 2B** **)**, migration **(** **Figure 2C** **)**, proliferation and cell viability **(** **Figure 2D** **)** of HTR-8 cells under HG exposure while slight or no variation was observed in the control group (Si-NC-normal *vs*. Si-TIGAR-normal). As they are basic elements necessary for ensuring the successful remodeling of maternal-fetal interface and the completion of pregnancy, therefore we concluded that HG exposure aggravated TIGAR-knockdown induced trophoblasts’ impairment on viability and biological function. Moreover, an oxidative stress environment in TIGAR deficient cells in HG condition was observed, accompanied with excessive leakage of LDH, decreased levels of SOD and GSH, reduced ratio of NADPH to NADP^+^ **(** **Figure 2E** **)**. Then we detected intracellular ROS production with DCFH-DA staining, as shown in **Figure 2F, G**, abundant ROS was generated with accompanied by a clear decrease in TIGAR expression, especially when cells were cultured in HG medium, detailly, a similar change was observed at the fluorescence intensity of MitoSox-Red **(** **Figure 2H** **)**, which indicated that mitochondria were the main source of ROS leakage. In addition, we observed increasing JC-1 monomer (green) and decreasing JC-1 aggregates (red), suggesting the dissipation of mitochondrial membrane potential and disrupted mitochondrial integrity upon TIGAR loss in HG exposure **(** **Figure 2I** **)**. Overall, loss of TIGAR impairs trophoblasts’ normal function and induces excess ROS damage under HG condition which will result in placental dysfunction.

### TIGAR knockdown induces trophoblast pyroptosis upon HG stimulation

**Figure 3:**
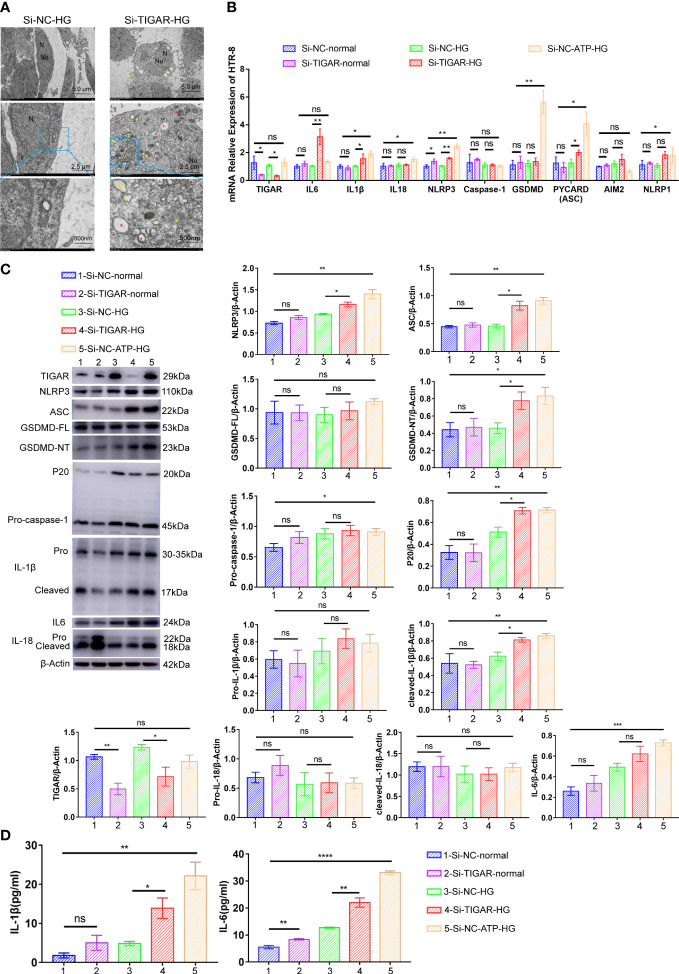
TIGAR deficiency induced pyroptosis in HTR-8 upon HG treatment. **(A)** Representative transmission electron microscopy (TEM) images of HTR-8 cells when cultured in normal or HG condition, which was transfected with Si-TIGAR or Si-NC. Red arrowheads, membrane pores and disrupted regions; Yellow arrowheads, large bubbles; Red arrow, overt mitochondria swelling; Red asterisks*, double-membrane autophagosomes (AP); Yellow asterisks *, autolysosomes (ASS). Expression of NLRP3, ASC, GSDMD-FL, GSDMD-NT, Pro-caspase-1, P20, Pro-IL-1β, cleaved-IL-1b, and IL-6 in HTR-8 cells, as detected on RNA **(B)** and protein levels **(C)**. The expression of these proteins is quantified by normalizing to β-actin *(n=3)*. **(D)** ELISA for IL-1β and IL-6 release of HTR-8 cells treated with normal/HG in the presence or absence of TIGAR knockdown. Data of the triple experiments are presented as the means ± SEM. *p < 0.05, **p < 0.01, ***p < 0.001, ****p < 0.0001 versus the indicated group, ns, no significance.

Next, we evaluated the characterization and cytotoxicity of HTR-8 cells after TIGAR knockdown in high glucose. The physicochemical properties of HTR-8 cells with different gene editing were characterized through TEM. As shown in **Figure 3A**, typical cytoplasm, nuclei (N) and nucleolus (Nu) were presented in the control group; however, in TIGAR knockdown group, TEM images revealed the cell surface lacked complex features and contained membrane pores and disrupted regions (red arrowheads). Yellow arrowheads indicated the large bubbles emerging from the plasma membrane which were canonical pyroptosis phenotype. And in the cytoplasm, overt mitochondria swelling (red arrow) and double-membrane autophagosomes (AP, *red asterisks) were observed. Notably, darkly stained autolysosomes indicated that autolysosomes (ASS, *yellow asterisks) were activated upon TIGAR knockdown.

To reveal the underlying mechanism of TIGAR deficiency induced adverse effects on HTR-8, we detected the expression of key molecules involved in pyroptosis. As shown in **Figures 3B–D**, compared with the control group (normal, 5.5mM), the expression of NLRP3, ASC, GSDMD, Caspase-1, IL-1β, IL-18 and IL-6 were upregulated after ATP treatment, and the majority of these proteins showed an increasing trend after TIGAR knockdown when cultured in high glucose medium, while the diversity was slight under normal glucose(5.5mM) treatment. The elevated expression of GSDMD-NT (P30 fragment of GSDMD) verified the pores generated during pyroptosis. Therefore, loss of TIGAR induces a pro-inflammatory response and trophoblast pyroptosis under high glucose stimulation.

### Pyroptosis of TIGAR-downregulated trophoblasts depends on the assembling of NLRP3 inflammasome

**Figure 4:**
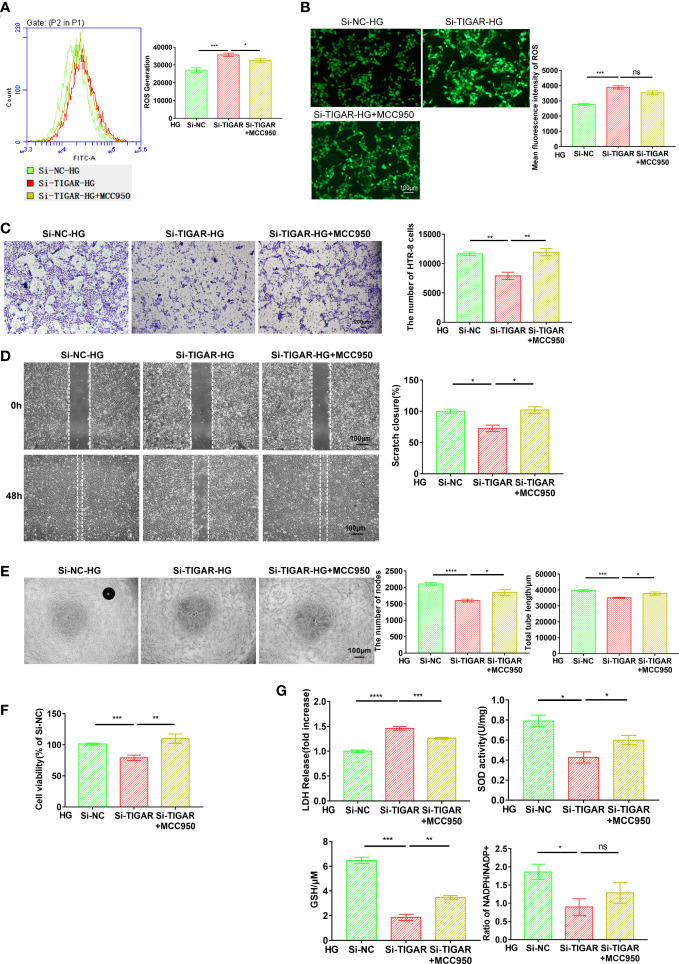
Loss of TIGAR mediated pyroptosis depends on the assembling of NLRP3 inflammasome. The detection of intracellular ROS in Si-TIGAR and Si-NC HTR-8 cells, which were pre-treated with MCC950 for 1 h then specific medium were added, as measured by flow cytometry **(A)** and photograph **(B)**. The histogram shows the mean ROS production. Scale bar: 100μm. The evaluation of cell function, as detected by cell invasion **(C)** (Scale bar: 200μm), wound scratch assay **(D)** (Scale bar: 100μm), and tube formation **(E)** (Scale bar: 100μm). **(F)** CCK-8 assay showing the alteration of cell viability between groups *(n=3)* **(G)** LDH release, total SOD activity, GSH contents, ratio of NADPH/NADP^+^ were measured after indicated treatment. Data of the triple experiments are presented as the means ± SEM. *p < 0.05, **p < 0.01, ***p < 0.001, ****p < 0.0001 versus the indicated group, ns, no significance.

Since multiple routes leads to pyroptosis, we examined the role of NLRP3 in TIGAR knockdown induced pyroptosis. After suppressing NLRP3 using specific inhibitor-MCC950, the cell behavior [like cell invasion **(** **Figure 4C** **),** migration **(** **Figure 4D** **)** and tube formation **(** **Figure 4E** **)**], cell viability **(** **Figure 4F** **)** and the severe oxidative stress **(** **Figure 4G** **)**, which were impaired by the deficiency of TIGAR, were significantly revised or even enhanced. Nevertheless, the generation and accumulation of ROS were slightly upregulated with no obvious variance in MCC950+HG group **(** **Figures 4A, B** **)**.

**Figure 5:**
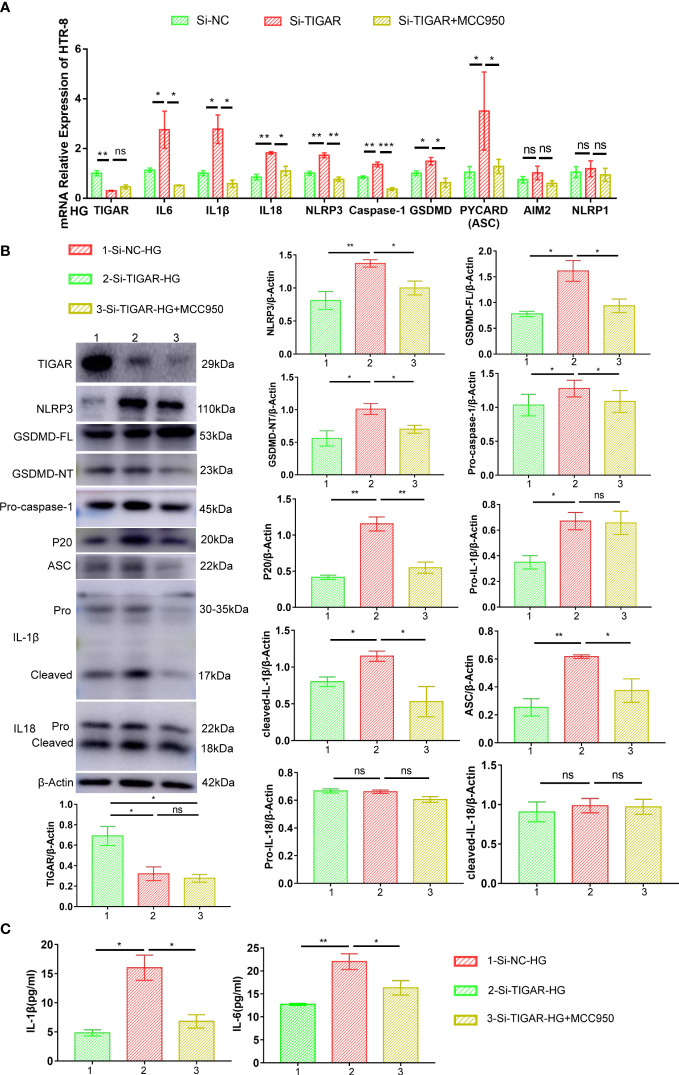
MCC950 treatment hindered the development of inflammation and pyroptosis. Real-time PCR **(A)** and western blotting **(B)** was used to analyze the expression of proinflammatory cytokines (Pro-IL-1β, cleaved-IL-1β, IL18 and IL-6) and pyroptosis factors (NLRP3, ASC, GSDMD-FL, GSDMD-NT, Pro-caspase-1, P20). The expression of these proteins was quantified by normalizing to β-actin (*n=3*) (right). **(C)** IL-1β and IL-6 in the supernatant were measured by ELISA. Data of the triple experiments are presented as the means ± SEM. *p < 0.05, **p < 0.01, ***p < 0.001 versus the indicated group, ns, no significance.

Furthermore, the expression of NLRP3, along with NLRP3-dependent caspase-1 activation, ASC, P20, IL-18, and IL-1β maturation, were downregulated by MCC950 treatment, as compared with untreated TIGAR deficient cells **(** **Figures 5A, B** **)**. The inhibitory effect of MCC950 on the maturation and release of IL-1β and IL-6 was further confirmed by ELISA **(** **Figure 5C** **)**. Besides, the expression of NLRP1 and AIM2 at mRNA level was detected for verificating the specificity of NLRP3-induced pyroptosis, consistently, there was no statistically change in the expression of both genes **(** **Figure 5A** **)**. These results demonstrate that exposure of TIGAR-deficient trophoblasts to HG induces assembly of NLRP3 inflammasome and leads to pyroptosis.

### ROS elimination ameliorates the suppressive effect of TIGAR deficiency on trophoblast’ function

**Figure 6:**
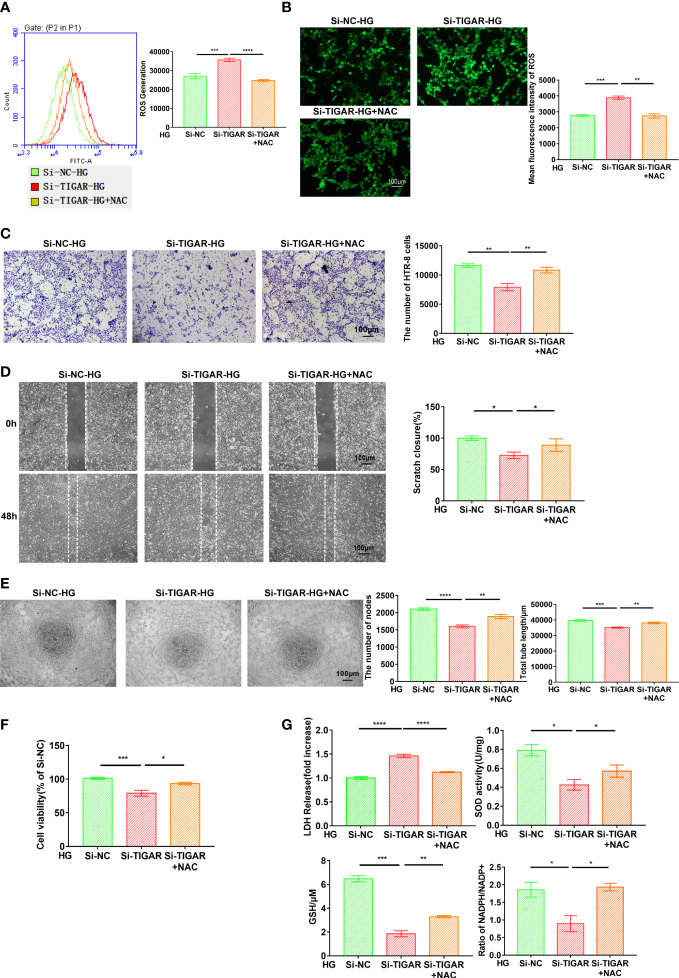
NAC attenuated oxidative stress and ameliorated cell function generated due to TIGAR deficiency. After successfully infected with TIGAR-siRNA or NC-siRNA, HTR-8 cells were pretreated with NAC for 1h and then cultured in HG condition prior to further experiments. Intracellular ROS levels were stained by DCFH-DA and then evaluated through flow cytometry **(A)** and monitored using a fluorescence microscope **(B)**. Scale bar, 100 μm. Cell migration **(C)**, invasion **(D)**, tube formation **(E)** and cell viability **(F)** were assessed at the indicated times. **(G)** The evaluation of LDH release, SOD activity, GSH levels and NADPH/NADP^+^ when cells pretreated with indicated concentration of NAC. Data of the triple experiments are presented as the means ± SEM. *p < 0.05, **p < 0.01, ***p < 0.001, ****p < 0.0001 versus the indicated group.

It is well known that TIGAR play an essential role in the contribution of ROS limitation, the foregoing evidence **(** **Figures 2F, G** **)** also verified that TIGAR deficiency induced ROS leakage. Subsequently, we lowered ROS level by treatment with the antioxidant N-acetyl-L-cysteine (NAC), as was detected by flow cytometry **(** **Figure 6A** **)** and immunofluorescence **(** **Figure 6B** **)**. The abilities of cell invasion **(** **Figure 6C** **)**, migration **(** **Figure 6D** **)**, tube formation **(** **Figure 6E** **)** and cell viability **(** **Figure 6F** **)** were significantly reversed by NAC treatment. Thus, NAC could inhibit cell viability loss and cytotoxicity induced by TIGAR loss in trophoblasts. As expected, NAC could also remarkably alleviate the excessive oxidative stress **(** **Figure 6G** **)**. Based on the foregoing evidence, we conclude that NAC behaves a protective role in cell function and limits the generation and accumulation of ROS, so that scavenging ROS by antioxidant peptide may be beneficial in the setting of abnormal glucose metabolism.

**Figure 7:**
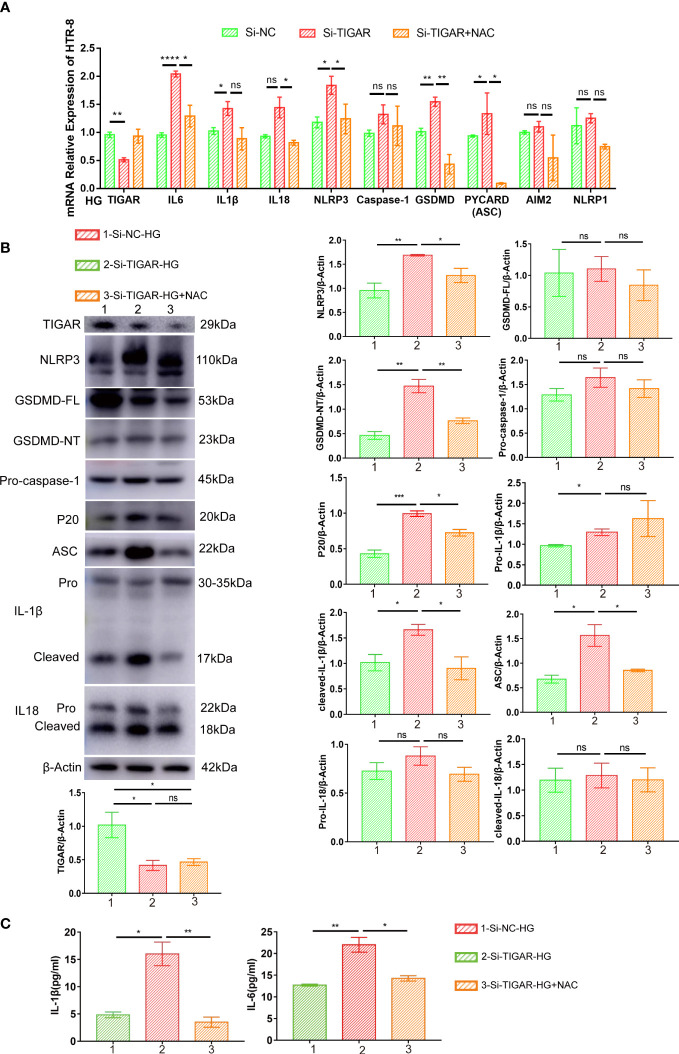
NAC alleviated pyroptosis induced by TIGAR knockdown in HG condition. The expression of proinflammatory cytokines (Pro-IL-1β, cleaved-IL-1β, IL18 and IL-6) and pyroptosis factors (NLRP3, ASC, GSDMD-FL, GSDMD-NT, Pro-caspase-1, P20) was evaluated by real-time PCR **(A)** and western blot **(B)**. β-actin were used as internal control. Bar graphs depict semi-quantitative analysis of protein expression levels. Dynamic changes of IL-1β and IL-6 were assessed by ELISA. Data of the triple experiments are presented as the means ± SEM. *p < 0.05, **p < 0.01, ***p < 0.001, ****p < 0.0001 versus the indicated group, ns, no significance.

To confirm the contribution of NAC in protecting pyroptosis, we evaluated the associated pathway and inflammatory cytokines. As **Figure 7** showed, elevation of IL-6, IL-1β, IL-18 were diminished in NAC group **(** **Figures 7A–C** **)** and pyroptosis induced by TIGAR deficiency was also improved, as evidenced by reduced expression of classic pyroptosis markers, including GSDMD-NT, NLRP3, ASC, and cleaved caspase-1(P20) **(** **Figures 7A, B** **).** Taken together, these data indicate that ROS elimination significantly inhibited the activation of NLRP3 inflammasome and NLRP3-mediated pyroptosis in TIGAR-deficient trophoblasts under HG condition.

## Discussion

Many studies in various models have indicated that TIGAR plays a role in ROS limitation and ROS promotion which drives disease initiation and development. As upregulated TIGAR was modulated both *in vivo* and *in vitro*, we assumed that TIGAR functioned as a compensatory mechanism to defend against oxidative stress. The present study demonstrated that TIGAR deficiency significantly increased the expression of NLRP3 inflammasome-related proteins, which subsequently led to pyroptosis in HG condition. Moreover, these effects were blocked by receptor inhibition (MCC950) and ROS elimination (NAC), indicating exacerbation of pyroptosis in TIGAR deficient trophoblasts is probably caused by NLRP3 inflammasome activation and excessive oxidative stress. Consistent with other studies, we found that loss of TIGAR could suppress the viability and proliferation of trophoblasts. Data obtained in this study have a significant meaning in the evaluation of TIGAR regulation and contribute to the prevention and treatment of related diseases.

Presence of hypoxia and metabolic disturbance give rise to oxidative stress, which is characterized by formation of large amounts of ROS, yielding several pregnancy complications, including preeclampsia, fetal growth restriction, GDM and even abortion (43, 44). As a highly reactive radicals, ROS damaged cell through inducing lipid peroxidation, protein and amino acid modifications, and DNA oxidation. TIGAR has been shown to support flux through the oxidative PPP, which generates NADPH for antioxidant defense. By this means, TIGAR exhibits an anti-pyroptotic effect by reducing the ROS levels. In this study, we demonstrated that TIGAR deficiency attacked trophoblasts in HG condition by reducing the PPP flux and elevating ROS levels, manifested as upregulated fluorescence intensity of mitochondria-specific ROS indicator MitoSox-Red and alteration of mitochondrial membrane potential. In short, our results show that TIGAR limits oxidative stress, a function that correlates with the ability of TIGAR to support the initial stages of fetal development. To investigate the role of ROS in NLRP3-mediated pyroptosis, we removed intracellular ROS with NAC and observed reduced expression of pyroptotic molecules. Based on the foregoing evidence, we speculate that ROS elevation is the first signal of NLRP3 inflammasome, and the autophagy or even the mitophagic signaling could play a vital role in mtROS-amplified NLRP3 inflammasome assembly and final activation, which is the second signal. Thus, the autophagy and alteration of mitochondrial membrane potential need urgent discussion in the following research.

GSDMD is an newly identified emerging target in the downstream of pyroptosis. Activation of NLRP3 inflammation leads the cleavage of full-length GSDMD to generate the GSDMD N-terminus (P30 fragment) as the molecule of pyroptosis (45). Activated GSDMD-N embed into the cell membrane for the formation of oligomeric pores, which caused the release of proinflammatory cytokines and changes of osmotic pressures, resulting in cell swelling and rupture ultimately (46, 47). Notably, ATP-induced pyroptosis elevated the expression of GSDMD at RNA and protein levels, while we merely discovered cleaved GSDMD protein increased upon TIGAR knockdown. Based on the foregoing evidence, we speculate that compared to ATP, TIGAR is a mild regulatory element in pyroptosis construction.

Inflammation and oxidative stress response in placenta play crucial roles in GDM. We detected elevated oxidative damage and inflammatory cytokines with the release of IL-18, IL-1β and IL-6, *via* GSDMD pore upon TIGAR loss, which was commonly considered as the terminal events of pyroptosis. Functionally, IL-1β was produced by nodlike receptor (NLR) protein inflammasomes upon many disorders, especially in the vasodilation and immune cell extravasation, which could sense “danger- or damage-associated molecular pattern (DAMPs) and amplify the inflammatory signals substantially (48, 49). IL-18, structurally similar to IL-1β, is a member of IL-1 superfamily of cytokines, has an important role in promoting the secretion of IL-1β and trigger the inflammatory storm *in vivo (* 50). IL-6 plays a detrimental role due to its association in the initiation and development of inflammatory disease, diabetic cardiomyopathy, and lung dysfunction (51, 52). As the inflammatory cascade progresses, IL-1β induce the secretion of additional NLRP3 cytokines such as IL-6 which can subsequently be observed in the supernatant and internal circulatory system and function as secondary inflammatory mediator (35, 36). Combined with the elevated levels of proinflammatory cytokines both within the cell and in cell supernatant, herein we provided convincing evidence for the initiation of trophoblast pyroptosis as induced by TIGAR deficiency under HG condition.

To sum up, we firstly informed the alterations on TIGAR-induced under HG condition, such as the morphology of trophoblasts, the appearance of intracellular vacuoles, and the activation of signaling pathways that promote migration and invasion, and explored comprehensively the biological function of TIGAR in oxidative stress limitation and pyroptosis prevention, which further suggested a causal relationship between HG exposure, ROS elevation, NLRP3 inflammasome assembly, and pyroptosis. Notably, our study underscores the multifaceted role of ROS in controlling GDM progression, intracellular and mitochondrion ROS were both demonstrated its importance in NLRP3-ASC inflammasome assembly. Although the response to ROS may reflect the overall level of oxidative stress during GDM progression, previous studies have shown that different ROS species, or different locations of ROS accumulation within cells, can convey differential effects on proliferation and survival (53, 54). Moreover, TIGAR was shown to more effectively limit mitochondrial than cytosolic ROS (55). Therefore, different cell’s ability in respond to ROS, a subtler contribution of different types of ROS and the precise mechanism in the limitation of mitochondrial ROS by TIGAR remain to be determined. These data determine the roles of different antioxidants and therapies in manipulation of ROS and the timing/sequence of combined treatments involving ROS which highlight an emerging insight into the mechanistic study and clinical treatment of GDM and other inflammatory diseases, as TIGAR conserves antioxidative, anti-inflammatory and pyroptosis prevention effects.

## Data availability statement

The raw data supporting the conclusions of this article will be made available by the authors, without undue reservation.

## Ethics statement

The studies involving human participants were reviewed and approved by the ethics committee of Maternal and Child Health Care Hospital of Shandong Province Affiliated to Qingdao University. The patients/participants provided their written informed consent to participate in this study. The animal study was reviewed and approved by the ethics committee of Maternal and Child Health Care Hospital of Shandong Province Affiliated to Qingdao University.

## Author contributions

JG performed most of experiments and drafted manuscript. MJZ analysed the data. MZ executed animal experiments. SL and ZF collected the clinical samples. AL and MHZ designed the study. AL supervised experiments. MHZ secured funding. AL and MHZ contributed equally to this work. All authors contributed to thearticle and approved the submitted version.

## References

[1] Weiss U Cervar M Puerstner P Schmut O Haas J Mauschitz R. Hyperglycaemia *in vitro* alters the proliferation and mitochondrial activity of the choriocarcinoma cell lines BeWo, JAR and JEG-3 as models for human first-trimester trophoblast. Diabetologia (2001) 44(2):209–19. doi: 10.1007/s001250051601 11270678

[2] Lewandowska M. Gestational diabetes mellitus (GDM) risk for declared family history of diabetes, in combination with BMI categories. Int J Environ Res Public Health (2021) 18(13):6936. doi: 10.3390/ijerph18136936 34203509 PMC8293805

[3] Chen X Zhang Y Chen H Jiang Y Wang Y Wang D. Association of maternal folate and vitamin B(12) in early pregnancy with gestational diabetes mellitus: A prospective cohort study. Diabetes Care (2021) 44(1):217–23. doi: 10.2337/dc20-1607 PMC7783943 33158950

[4] Casagrande SS Linder B Cowie CC. Prevalence of gestational diabetes and subsequent type 2 diabetes among U.S. women. Diabetes Res Clin Pract (2018) 141:200–8. doi: 10.1016/j.diabres.2018.05.010 29772286

[5] Mustafa M Bogdanet D Khattak A Carmody LA Kirwan B Gaffney G. Early gestational diabetes mellitus (GDM) is associated with worse pregnancy outcomes compared with GDM diagnosed at 24-28 weeks gestation despite early treatment. QJM (2021) 114(1):17–24. doi: 10.1093/qjmed/hcaa167 32413109

[6] Tang X Pronk W Traber J Liang H Li G Morgenroth E. Integrating granular activated carbon (GAC) to gravity-driven membrane (GDM) to improve its flux stabilization: Respective roles of adsorption and biodegradation by GAC. Sci Total Environ (2021) 768:144758. doi: 10.1016/j.scitotenv.2020.144758 33454492

[7] Gomez Ribot D Diaz E Fazio MV Gómez HL Fornes D Macchi SB. An extra virgin olive oil-enriched diet improves maternal, placental, and cord blood parameters in GDM pregnancies. Diabetes Metab Res Rev (2020) 36(8):e3349. doi: 10.1002/dmrr.3349 32447799

[8] Du P Li X Yang Y Zhou Z Fan X Chang H. Regulated-biofilms enhance the permeate flux and quality of gravity-driven membrane (GDM) by *in situ* coagulation combined with activated alumina filtration. Water Res (2021) 209:117947. doi: 10.1016/j.watres.2021.117947 34910991

[9] Bensaad K Tsuruta A Selak MA Vidal MN Nakano K Bartrons R. TIGAR, a p53-inducible regulator of glycolysis and apoptosis. Cell (2006) 126(1):107–20. doi: 10.1016/j.cell.2006.05.036 16839880

[10] Green DR Chipuk JE. p53 and metabolism: Inside the TIGAR. Cell (2006) 126(1):30–2. doi: 10.1016/j.cell.2006.06.032 16839873

[11] Cheung EC DeNicola GM Nixon C Blyth K Labuschagne CF Tuveson DA. Dynamic ROS control by TIGAR regulates the initiation and progression of pancreatic cancer. Cancer Cell (2020) 37(2):168–182 e4. doi: 10.1016/j.ccell.2019.12.012 31983610 PMC7008247

[12] Mierzyński R Poniedziałek-Czajkowska E Sotowski M Szydełko-Gorzkowicz M. Nutrition as prevention factor of gestational diabetes mellitus: A narrative review. Nutrients (2021) 13(11):3787. doi: 10.3390/nu13113787 34836042 PMC8625817

[13] Zhang DM Zhang T Wang MM Wang XX Qin YY Wu J. TIGAR alleviates ischemia/reperfusion-induced autophagy and ischemic brain injury. Free Radic Biol Med (2019) 137:13–23. doi: 10.1016/j.freeradbiomed.2019.04.002 30978385

[14] Mittler R. ROS are good. Trends Plant Sci (2017) 22(1):11–9. doi: 10.1016/j.tplants.2016.08.002 27666517

[15] Srinivas US Tan BWQ Vellayappan BA Jeyasekharan AD. ROS and the DNA damage response in cancer. Redox Biol (2019) 25:101084. doi: 10.1016/j.redox.2018.101084 30612957 PMC6859528

[16] Yang B Chen Y Shi J. Reactive oxygen species (ROS)-based nanomedicine. Chem Rev (2019) 119(8):4881–985. doi: 10.1021/acs.chemrev.8b00626 30973011

[17] Cheung EC Vousden KH. The role of ROS in tumour development and progression. Nat Rev Cancer (2022) 22(5):280–97. doi: 10.1038/s41568-021-00435-0 35102280

[18] Ochoa CD Wu RF Terada LS. ROS signaling and ER stress in cardiovascular disease. Mol Aspects Med (2018) 63:18–29. doi: 10.1016/j.mam.2018.03.002 29559224 PMC6139279

[19] Ford K Hanley CJ Mellone M Szyndralewiez C Heitz F Wiesel P. NOX4 inhibition potentiates immunotherapy by overcoming cancer-associated fibroblast-mediated CD8 t-cell exclusion from tumors. Cancer Res (2020) 80(9):1846–60. doi: 10.1158/0008-5472.CAN-19-3158 PMC7611230 32122909

[20] Chakrabarty RP Chandel NS. Mitochondria as signaling organelles control mammalian stem cell fate. Cell Stem Cell (2021) 28(3):394–408. doi: 10.1016/j.stem.2021.02.011 33667360 PMC7944920

[21] Zhao M Wang Y Li L Liu S Wang C Yuan Y. Mitochondrial ROS promote mitochondrial dysfunction and inflammation in ischemic acute kidney injury by disrupting TFAM-mediated mtDNA maintenance. Theranostics (2021) 11(4):1845–63. doi: 10.7150/thno.50905 PMC7778599 33408785

[22] Schriner SE Linford NJ Martin GM Treuting P Ogburn CE Emond M. Extension of murine life span by overexpression of catalase targeted to mitochondria. Science (2005) 308(5730):1909–11. doi: 10.1126/science.1106653 15879174

[23] Sabharwal SS Schumacker PT. Mitochondrial ROS in cancer: initiators, amplifiers or an achilles' heel? Nat Rev Cancer (2014) 14(11):709–21. doi: 10.1038/nrc3803 PMC4657553 25342630

[24] Xue D Zhou X Qiu J. Emerging role of NRF2 in ROS-mediated tumor chemoresistance. BioMed Pharmacother (2020) 131:110676. doi: 10.1016/j.biopha.2020.110676 32858502

[25] Harris IS DeNicola GM. The complex interplay between antioxidants and ROS in cancer. Trends Cell Biol (2020) 30(6):440–51. doi: 10.1016/j.tcb.2020.03.002 32303435

[26] Fendt SM Lunt SY. Dynamic ROS regulation by TIGAR: Balancing anti-cancer and pro-metastasis effects. Cancer Cell (2020) 37(2):141–2. doi: 10.1016/j.ccell.2020.01.009 32049042

[27] Yu P Zhang X Liu N Tang L Peng C Chen X. Pyroptosis: mechanisms and diseases. Signal Transduct Target Ther (2021) 6(1):128. doi: 10.1038/s41392-021-00507-5 33776057 PMC8005494

[28] Tang R Xu J Zhang B Liu J Liang C Hua J. Ferroptosis, necroptosis, and pyroptosis in anticancer immunity. J Hematol Oncol (2020) 13(1):110. doi: 10.1186/s13045-020-00946-7 32778143 PMC7418434

[29] Kovacs SB Miao EA. Gasdermins: Effectors of pyroptosis. Trends Cell Biol (2017) 27(9):673–84. doi: 10.1016/j.tcb.2017.05.005 PMC5565696 28619472

[30] Shi J Gao W Shao F. Pyroptosis: Gasdermin-mediated programmed necrotic cell death. Trends Biochem Sci (2017) 42(4):245–54. doi: 10.1016/j.tibs.2016.10.004 27932073

[31] Zhaolin Z Guohua L Shiyuan and W. Zuo W. Role of pyroptosis in cardiovascular disease. Cell Prolif (2019) 52(2):e12563. doi: 10.1111/cpr.12563 30525268 PMC6496801

[32] Du T Gao J Li P Wang Y Qi Q Liu X. Pyroptosis, metabolism, and tumor immune microenvironment. Clin Transl Med (2021) 11(8):e492. doi: 10.1002/ctm2.492 34459122 PMC8329701

[33] Alehashemi S Goldbach-Mansky R. Human autoinflammatory diseases mediated by NLRP3-, pyrin-, NLRP1-, and NLRC4-inflammasome dysregulation updates on diagnosis, treatment, and the respective roles of IL-1 and IL-18. Front Immunol (2020) 11:1840. doi: 10.3389/fimmu.2020.01840 32983099 PMC7477077

[34] He Y Hara H Núñez G. Mechanism and regulation of NLRP3 inflammasome activation. Trends Biochem Sci (2016) 41(12):1012–21. doi: 10.1016/j.tibs.2016.09.002 PMC5123939 27669650

[35] Li Y Song W Tong Y Zhang X Zhao J Gao X. Isoliquiritin ameliorates depression by suppressing NLRP3-mediated pyroptosis *via* miRNA-27a/SYK/NF-κB axis. J Neuroinflamm (2021) 18(1):1. doi: 10.1186/s12974-020-02040-8 PMC7786465 33402173

[36] Grebe A Hoss F Latz E. NLRP3 inflammasome and the IL-1 pathway in atherosclerosis. Circ Res (2018) 122(12):1722–40. doi: 10.1161/CIRCRESAHA.118.311362 29880500

[37] Gou X Xu W Liu Y Peng Y Xu W Yin Y. IL-6 prevents lung macrophage death and lung inflammation injury by inhibiting GSDME- and GSDMD-mediated pyroptosis during pneumococcal pneumosepsis. Microbiol Spectr (2022) 10(2):e0204921. doi: 10.1128/spectrum.02049-21 35297653 PMC9045248

[38] Voet S Srinivasan S Lamkanfi and G. van Loo M. Inflammasomes in neuroinflammatory and neurodegenerative diseases. EMBO Mol Med (2019) 11(6):e10248. doi: 10.15252/emmm.201810248 31015277 PMC6554670

[39] Hanna J Goldman-Wohl D Hamani Y Avraham I Greenfield C Natanson-Yaron S. Decidual NK cells regulate key developmental processes at the human fetal-maternal interface. Nat Med (2006) 12(9):1065–74. doi: 10.1038/nm1452 16892062

[40] Zhao Y Jia X Yang X Bai X Lu Y Zhu L. Deacetylation of caveolin-1 by Sirt6 induces autophagy and retards high glucose-stimulated LDL transcytosis and atherosclerosis formation. Metabolism (2022) 131:155162. doi: 10.1016/j.metabol.2022.155162 35167876

[41] Yin Y Pan Y He J Zhong H Wu Y Ji C. The mitochondrial-derived peptide MOTS-c relieves hyperglycemia and insulin resistance in gestational diabetes mellitus. Pharmacol Res (2022) 175:105987. doi: 10.1016/j.phrs.2021.105987 34798268

[42] Zhou Y Zhao R Lyu Y Shi H Ye W Tan Y. Serum and amniotic fluid metabolic profile changes in response to gestational diabetes mellitus and the association with maternal-fetal outcomes. Nutrients (2021) 13(10):3644. doi: 10.3390/nu13103644 34684645 PMC8539410

[43] Chiarello DI Abad C Rojas D Toledo F Vázquez CM Mate A. Oxidative stress: Normal pregnancy versus preeclampsia. Biochim Biophys Acta Mol Basis Dis (2020) 1866(2):165354. doi: 10.1016/j.bbadis.2018.12.005 30590104

[44] Hu C Wu Z Huang Z Hao X Wang S Deng J. Nox2 impairs VEGF-a-induced angiogenesis in placenta *via* mitochondrial ROS-STAT3 pathway. Redox Biol (2021) 45:102051. doi: 10.1016/j.redox.2021.102051 34217063 PMC8258686

[45] Wang K Sun Q Zhong X Zeng M Zeng H Shi X. Structural mechanism for GSDMD targeting by autoprocessed caspases in pyroptosis. Cell (2020) 180(5):941–955 e20. doi: 10.1016/j.cell.2020.02.002 32109412

[46] Wang Y Shi P Chen Q Huang Z Zou D Zhang J. Mitochondrial ROS promote macrophage pyroptosis by inducing GSDMD oxidation. J Mol Cell Biol (2019) 11(12):1069–82. doi: 10.1093/jmcb/mjz020 PMC6934151 30860577

[47] Barnett KC Ting JP. Mitochondrial GSDMD pores DAMPen pyroptosis. Immunity (2020) 52(3):424–6. doi: 10.1016/j.immuni.2020.02.012 PMC7337261 32187511

[48] Schneider KS Groß CJ Dreier RF Saller BS Mishra R Gorka O. The inflammasome drives GSDMD-independent secondary pyroptosis and IL-1 release in the absence of caspase-1 protease activity. Cell Rep (2017) 21(13):3846–59. doi: 10.1016/j.celrep.2017.12.018 PMC5750195 29281832

[49] Karmakar M Minns M Greenberg EN Diaz-Aponte J Pestonjamasp K Johnson JL. N-GSDMD trafficking to neutrophil organelles facilitates IL-1β release independently of plasma membrane pores and pyroptosis. Nat Commun (2020) 11(1):2212. doi: 10.1038/s41467-020-16043-9 32371889 PMC7200749

[50] Miao N Yin F Xie H Wang Y Xu Y Shen Y. The cleavage of gasdermin d by caspase-11 promotes tubular epithelial cell pyroptosis and urinary IL-18 excretion in acute kidney injury. Kidney Int (2019) 96(5):1105–20. doi: 10.1016/j.kint.2019.04.035 31405732

[51] Xuemei G Wenchun X Yusi L Yang P Wenlong X Yibing Y. IL-6 prevents lung macrophage death and lung inflammation injury by inhibiting GSDME- and GSDMD-mediated pyroptosis during pneumococcal pneumosepsis. Microbiol Spectr (2022) 10(2):e0204921. doi: 10.1128/spectrum.02049-21 35297653 PMC9045248

[52] Tanaka T Narazaki M Kishimoto T. IL-6 in inflammation, immunity, and disease. Cold Spring Harb Perspect Biol (2014) 6(10):a016295. doi: 10.1101/cshperspect.a016295 25190079 PMC4176007

[53] Liou GY Döppler H DelGiorno KE Zhang L Leitges M Crawford HC. Mutant KRas-induced mitochondrial oxidative stress in acinar cells upregulates EGFR signaling to drive formation of pancreatic precancerous lesions. Cell Rep (2016) 14(10):2325–36. doi: 10.1016/j.celrep.2016.02.029 PMC4794374 26947075

[54] Dai DF Chen T Szeto H Nieves-Cintrón M Kutyavin V Santana LF. Mitochondrial targeted antioxidant peptide ameliorates hypertensive cardiomyopathy. J Am Coll Cardiol (2011) 58(1):73–82. doi: 10.1016/j.jacc.2010.12.044 21620606 PMC3742010

[55] Dodson M Darley-Usmar V Zhang J. Cellular metabolic and autophagic pathways: traffic control by redox signaling. Free Radic Biol Med (2013) 63:207–21. doi: 10.1016/j.freeradbiomed.2013.05.014 PMC3729625 23702245

