# Supplementary figures and images for: TIGAR deficiency induces caspase-1-dependent trophoblasts pyroptosis through NLRP3-ASC inflammasome
Source: Front Immunol. 2023 Apr 14;14:1114620. doi: 10.3389/fimmu.2023.1114620 (PMC10140348; doi:10.3389/fimmu.2023.1114620)

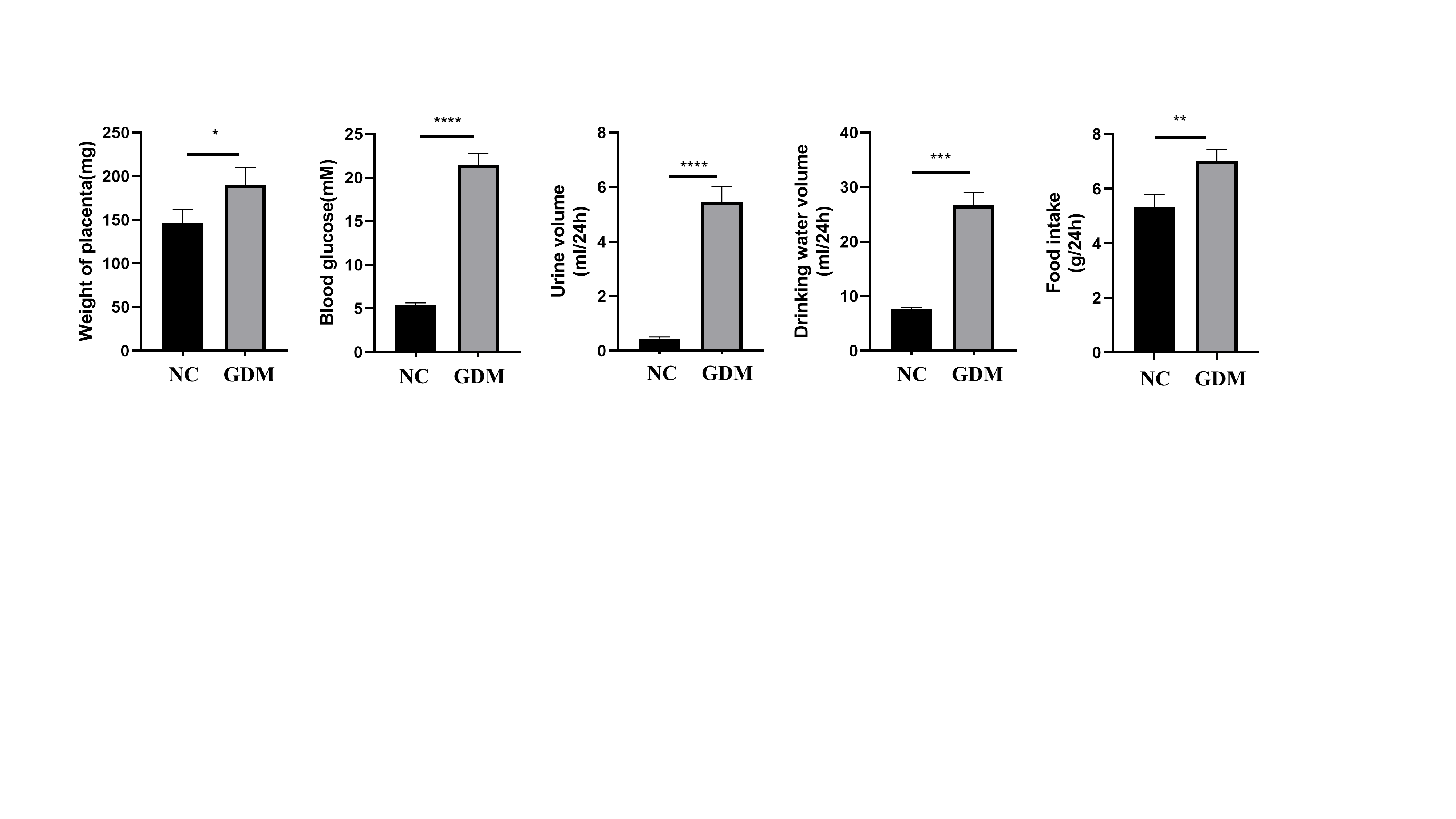

Supplement: Supplementary file 1 [file Image_1.tif]
